# Cyclodextrin-Based Nanoplatforms for Tumor Phototherapy: An Update

**DOI:** 10.3390/pharmaceutics14071375

**Published:** 2022-06-29

**Authors:** Xingjie Wu, Ying Chen, Qianqian Guo, Ling Tao, Yang Ding, Xianguang Ding, Xiangchun Shen

**Affiliations:** 1 The State Key Laboratory of Functions and Applications of Medicinal Plants, School of Pharmaceutical Sciences, Guizhou Medical University, University Town, Guian New District, Guiyang 550025, China; ychen1214@163.com (Y.C.); guoqqsci@126.com (Q.G.); tl15285581860@163.com (L.T.); 2 High Efficacy Application of Natural Medicinal Resources Engineering Center of Guizhou Province, School of Pharmaceutical Sciences, Guizhou Medical University, University Town, Guian New District, Guiyang 550025, China; 3 State Key Laboratory of Natural Medicines, Department of Pharmaceutics, China Pharmaceutical University, Nanjing 210009, China; dingypharm@cpu.edu.cn; 4 Key Laboratory for Organic Electronics and Information Displays & Jiangsu Key Laboratory for Biosensors, Nanjing University of Posts and Telecommunications, Nanjing 210023, China

**Keywords:** cyclodextrin, supermolecular assembly, photothermal therapy, photodynamic therapy

## Abstract

Tumor phototherapies are light-mediated tumor treatment modalities, which usually refer to tumor photothermal therapy (PTT) and photodynamic therapy (PDT). Due to the outstanding spatial-temporal control over treatment through light irradiation, tumor phototherapies display extremely low side effects during treatment and are believed to be a tumor treatment method with a clinical translation potential. However, current tumor phototherapy nanoplatforms face obstacles, including light irradiation-induced skin burning, tumor hypoxia microenvironments, limited light penetration depth, et al. Therefore, one important research direction is developing a tumor phototherapy nanoplatform with multifunctionality and enhanced pharmacological effects to overcome the complexity of tumor treatment. On the other hand, cyclodextrins (CDs) are starch-originated circular oligosaccharides with negligible toxicity and have been used to form supermolecular nanostructures through a host–guest interaction between the inner cavity of CDs and functional biomolecules. In the past few years, numerous studies have focused on CD-based multifunctional tumor phototherapy nanoplatforms with an enhanced photoeffect, responsive morphological transformation, and elevated drug bioavailability. This review focuses on the preparation methods of CD-based tumor phototherapy nanoplatforms and their unique physiochemical properties for improving anti-tumor pharmacological efficacy.

## 1. Introduction

Cancer phototherapy is a highly specific tumor treatment modality developed in the past decade. The two most intensively investigated cancer phototherapies are PTT and PDT, which are activated by light irradiation to generate heat or reactive oxygen species (ROS), respectively. For PTT, the generated heat can promote localized temperature elevation to cause cancer cell membrane disintegration and protein denaturation, which would further induce cell apoptosis and necrosis [1,2,3,4]. Such temperature elevation-induced cell inhibition can be dramatically intensified due to the insufficient circulating system inside tumor tissues. For PDT, the light irradiation-generated ROS can destroy subcellular structures, such as cell membrane and organelle membrane, by the rapid oxidation of biomolecules, in a localized manner [5,6,7], as ROS are extremely active with a short diffusion radio. More importantly, cancer phototherapy showed a synergistic effect with other cancer therapies by promoting a cargo release upon laser irradiation. For example, PTT-induced temperature elevation can accelerate a chemotherapy drug’s release by accelerating molecule diffusion within a nanoparticle [8,9]. Similarly, PDT-generated ROS can be applied as a drug release trigger when ROS-responsive materials are used to construct drug-loaded nanocarriers [10,11,12,13,14]. Owing to those unique features, both PTT and PDT were used to develop advanced anti-cancer nanoplatforms with low normal tissue toxicity and enhanced pharmacological efficacy.

**Figure 1:**
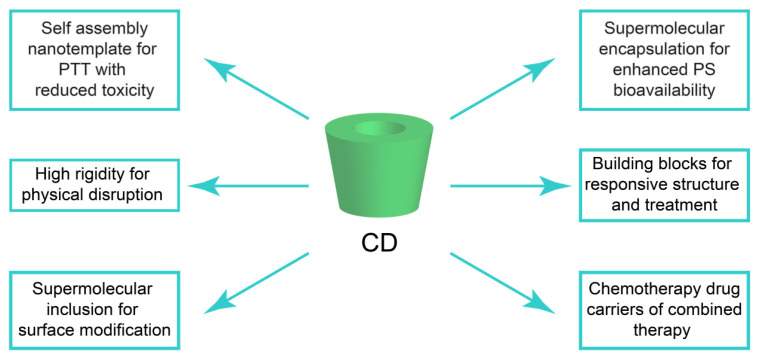
CD functionalities in tumor phototherapy nanoplatforms.

CDs are a kind of non-toxic and biocompatible cyclic-oligosaccharides consisting of α-1,4-glucosidic bonds linked to D-glucose [15]. Depending on the number of D-glucose unites, CDs have six, seven, and eight D-glucose units, respectively nominated as α-, β-, and γ-CD. CDs possess a rigid 3-D structure with a hydrophobic inner cavity, allowing the supermolecular inclusion of various biomolecules with appropriate size and hydrophobicity. In addition, the hydrophilic outside surface of CDs can endow the inclusion complex with water solubility. Therefore, CDs have been widely applied as a drug solubilizer for optimizing drug bioavailability and a building block for developing a hierarchy self-assembly nanostructure [16,17,18,19]. Nowadays, CDs and their derivative-based cancer phototherapy nanoplatforms are drawing greater attention in both academic and industrial societies. In this review, we first introduce the preparation of CD-based PTT and PDT nanoplatforms. Then, we discuss the roles CD played in those nanoplatforms and the mechanism underlying their unique physicochemical performance (Figure 1). Finally, the in vivo anti-tumor performance of CD-based nanoplatforms is evaluated to elucidate their advantages and challenges in future clinical translations.

## 2. CD-Based Nanoplatforms for PTT and Its Combined Therapies

Photothermal agents (PTAs) refer to materials capable of converting light to heat and are the key functional component of PTT nanoplatforms [3,20]. As the light at near infra-red I (NIR-I, 750–850 nm) and NIR-II (900–1200 nm) regions are considered safe windows for clinical treatment by the US Food and Drug Administration, various materials, including gold nanomaterials, 2-D materials, carbon nanomaterials, and conducting polymers were heavily investigated as NIR-I and/or NIR-II PTAs for anti-tumor PTT [21,22,23,24]. Note that the NIR-II region is believed to be less of an irritant and more transparent than the NIR-I region; as a result, PTAs with high NIR-II photothermal conversion efficiency (PCE) are drawing more and more attention. However, the clinical translations of current PTT technologies were severely impeded by the following factors: 1. The non-biocompatible nanotemplate and toxic surfactant used during PTA synthesis; 2. A light overdose due to an insufficient PCE; 3. A lack of multifunctionality. In this section, we introduce the synthesis and preparation of CD/PTA hybridization and discuss how CD/PTA hybridization nanostructures overcame the aforementioned problems (Table 1).

### 2.1. CD-Based Supermolecular Nanostructure as a Functional Nanotemplate for PTA

Non-spherical gold nanomaterials, such as gold nanoshells, gold nanorods (AuNR), and gold nanotriangles, were intensively applied as PTAs due to their localized surface plasmon resonance (LSPR) peak at the NIR light region [3,4,21]. As gold nanomaterials’ PCE was correlated with their morphology, CD-based self-assembly with a predesigned nanostructure can be applied as a biocompatible nanotemplate to enhance the PCE. For example, Wu et al. synthesized biodegradable poly(ethylene glycol)-b-polycysteine (PEG-b-PC) and further prepared polyrotaxane-b-PC micelle through the supermolecular interaction between the PEG segment and α-CD [25]. As polyrotaxane is highly hydrophobic, a PC segment with abundant thiol moieties was located at the micelle surface. Using this biocompatible micelle as a nanotemplate, thickened gold nanoshells with an elevated PCE can be formed through the strong thiol-Au complexation. In another report, a nitroaniline type NO generator-tethered β-CD was copolymerized with β-CD and epichlorohydrin. Under 420 nm light irradiation, the tethered NO generator can produce an NO capable of reducing Au^3+^ to Au^0^ to form gold nanotriangles or nanoflowers in situ [26]. This method provided a biofriendly synthesis route without gold seed, toxic reductant, and surfactant. The aggregation of small gold nanoparticles can red-shift its LSPR peak to induce photothermal conversion in the NIR region. Inspired by this, Yu et al. developed a pH-responsive PTT nanoplatform using α-CD as the steric hindrance. This nanoplatform consisted of two gold nanoparticles (AuNP) separately decorated with a pair of complementary DNA sequences at their surface. The 3′-end of the DNA sequence was covalently modified with α-CD through a pH-responsive linker, pyridine-2-imine. The conjugated α-CD can act as a steric hindrance to prevent AuNP’s aggregation in a neutral physiological environment, while a large AuNP aggregation with a high PCE at the NIR region can be in situ formed in acid tumor microenvironments (TMEs) due to the release of α-CD from AuNP’s surface [27]. The self-fabricated gold aggregation can also enhance an in vivo photoacoustic signal by a 3.5-fold and keep a high signal level within 24 h.

**Figure 2:**
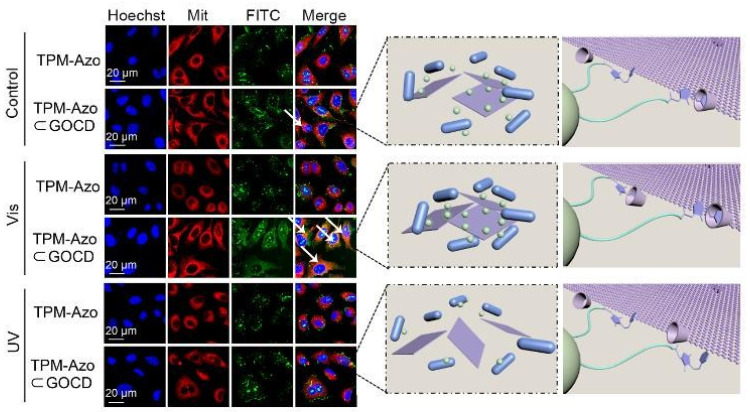
Light-controlled intracellular mitochondrial disruption by azobenzene and mitochondrion-targeting peptide-modified transferrin and CD-modified GO. Mitochondrial disruption (white arrows) after visible-light irradiation (Vis, 520 nm) but not after UV irradiation (UV, 365 nm). The effect of azobenzene and mitochondrion-targeting peptide-modified transferrin and CD-modified GO on intracellular mitochondrial dynamics was investigated by confocal microscopy. (Adapted with permission from Ref. [28]. Copyright 2019, Royal Soc. Chemistry).

A CD-based self-assembly nanostructure was reported to have shape transformation abilities under the help of stimuli-responsive moieties. Due to the structural rigidity of CD, the shape transformation of CD-based self-assembly can physically disrupt the subcellular structure. Zhang et al. modified the tumor-targeting protein transferrin with the mitochondrion-targeting peptide and PEG, whose terminals were further linked with azobenzene. After being bound with a cancer cell mitochondrion through a mitochondrion-targeting peptide, the light-responsive azobenzene at the transferrin’s surface can be used as a guest to form a supermolecular inclusion with β-CD-modified graphene oxide (GO) under 520 nm light irradiation (Figure 2). The in situ supermolecular assembly further caused mitochondrion aggregation and mitochondrial dysfunction, which significantly promoted the pharmacological efficacy of GO-based PTT against the S180 tumor-bearing mice [28]. In a similar work, a mitochondrion-targeting peptide-tethered magnetic nanoparticle and lipoic acid-modified AuNR were hybridized with each other through an amidation reaction. Under the guidance of a geomagnetic field, the magnetic nanoparticle-AuNR hybrid nanostructure and β-CD-modified hyaluronic acid can self-assemble to form photothermal nanofibril along the direction of the magnetic field, which can physically damage the cancer cell’s mitochondrion and clear the A549 cell for suppressed tumor metastasis after a PTT treatment [29].

### 2.2. CDs as Drug Carriers for Combined PTT and Chemotherapy

PTA, such as AuNR, magnetic nanoparticles, CuS nanocrystals, et al. are solid nanomaterials lacking the ability to encapsulate anti-cancer drugs. Thus, the CDs were coated at their surface to load the anti-cancer drugs through a host–guest interaction [30,31,43,44,45,46,47]. Furthermore, CD-encapsulated drugs can display a photothermal-triggered release due to the localized temperature elevation during PTT treatment. In one recent report, a magnetic nanoparticle was in situ-synthesized at GO’s surface and was further covalently modified with β-CD and cholic acid-modified hyaluronic acid. Then, the anti-cancer drug, camptothecin, was loaded at a high loading capacity of 442 mg/g by adsorbing at GO’s surface through π-π interaction and encapsulating within the β-CD cavity. As graphene and its derivatives are excellent NIR light PTAs, this hybrid GO nanoplatform displayed a photothermal-triggered camptothecin release profile and enhanced an anti-tumor efficacy under NIR light irradiation [30]. To further endow nanoparticles with a multi-responsive release profile, another research group grafted β-CD onto the HA chain through a pH- responsive linker, boronic acid. After covalently decorating β-CD at the fluorescent polydopamine nanoparticles’ surface, the anti-cancer drug paclitaxel was loaded into the β-CD cavity through a host–guest interaction. This multifunctional polydopamine nanoparticle can achieve a pH and NIR light dual-stimuli-triggered drug release and fluorescence imaging-guided chemo-photothermal therapy [31].

**Figure 3:**
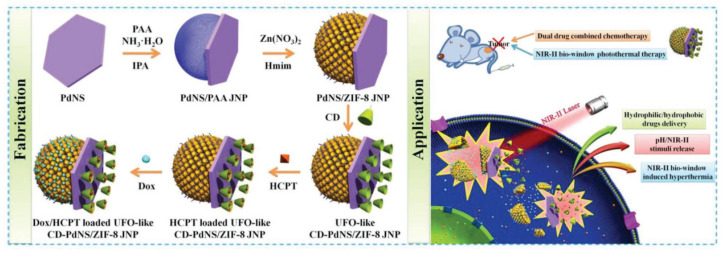
Schematic diagram of preparing the Janus nanoparticle for an in vitro and in vivo pH and NIR-II (1064 nm) dual-triggered synergistic dual-drug chemotherapy and photothermal therapy in the NIR-II biowindow. (Adapted with permission from Ref. [32]. Copyright 2018, Wiley).

Simultaneously loading two anti-cancer drugs with opposite hydrophilicity in a single nanocarrier has been an extremely difficult task for the traditional drug delivery system. To overcome this, drugs were loaded within different sectors of a CD-based Janus nanostructure for programmed release [32,48]. Zhang et al. selectively decorated one side of a palladium nanosheet with the zeolitic imidazolate framework-8 for loading the hydrophobic anti-cancer drug, 10-hydroxycamptothecin (Figure 3), while the other side was decorated with β-CD for loading the hydrophilic drug, doxorubicin (DOX). The obtained Janus nanoparticle displayed a pH and photothermally-triggered dual-drug release profile and enhanced in vivo anti-tumor therapeutic efficacy [32]. In addition, the palladium nanosheet displayed strong absorbance at the NIR-II region and endowed this hybrid nanoplatform with a deep tumor treatment potential.

Despite the photothermal effect-triggered drug release, combined PTT and chemotherapy still face a drug leaky release due to a large dilution volume in the circulation system. Therefore, premature drug release remains one major obstacle to highly specific and efficient tumor treatments. To fight against this, CDs or CD-modified PTAs were used to cap the pores of the drug-loaded mesoporous nanoparticle as the gatekeepers [33,49,50]. Compared to gold nanomaterials, mesoporous platinum nanoparticles are reported to have a stable NIR light PCE and enhanced biocompatibility. Inspired by this, Zhao et al. capped the pores of DOX-loaded mesoporous platinum nanoparticles with β-CD. As platinum can be oxidized to Pt^2+^ in the acid TME, these β-CD-capped mesoporous platinum nanoparticles exhibited both a photothermal effect and acid TME-triggered DOX release with the minimum leaky release [33]. 

Organic PTAs are a type of small molecule with a high NIR light PCE and biocompatibility, which can be either encapsulated within the CD cavity or covalently linked to CD for elevated bioavailability and biosafety [34,35,51,52,53]. After covalently linked to β-CD, organic PTA quaterrylene bisimide exhibited enhanced photostability, NIR light absorptivity, and photoacoustic imaging ability. More importantly, the β-CD modification of quaterrylene bisimide was reported to promote its renal clearance pathway, which is believed to have reduced long-term side effects and minimized toxicity [34]. Due to the mechanically interlocked molecule structure of polyrotaxane, polyrotaxanes at the polymer micelles’ surface also showed a drug gatekeeper effect. To prepare this type of micelle, the ends of polyrotaxanes were, respectively, modified with organic PTA perylene diimide and a targeting ligand cyclic-RGD peptide as the stoppers. Due to the π-π interaction between perylene diimide, a core-shell structure with polyrotaxanes at the surface was formed. After being crosslinked by N-hydroxysulfosuccinimide ester-activated disulfide moieties, the micelle displayed a 24 h cumulative release of 61.6% for paclitaxel under NIR light and a 10 mM glutathione treatment, which was increased by 10-fold compared to micelle without any treatment (6.27% within 24 h) [35].

Compared with oral or intravenous administration, drug administration through an injectable hydrogel implant can have better control over drug biodistribution and prolonged drug retention in tumor tissue. Using CDs as crosslinkers or building blocks, injectable hydrogels with a sustained drug release profile can be further obtained for tumor treatment without repeated drug administration [36,37,38]. Recently, adamantane (AD)-modified DOX was supermolecularly conjugated to the methacrylated poly-β-CD macromer, which was further used to form composite hydrogel with encapsulated AuNR through the radical copolymerization with N-isopropylacrylamide. The composite hydrogel displayed reversible drug release behavior due to the photothermally-induced hydrogel collapse and hydrogel swelling in a dark state [36]. Further in vivo experiments showed that hydrogel can achieve a 21-day DOX retention with a single hydrogel injection, which significantly reduced the discomfort of patients during treatment. 

### 2.3. CDs as Connecting Moieties for Sophisticated Surface Modification

In order to prepare a PDT nanoplatform with multifunctionality, sophisticated surface modifications were performed to construct nanolayers with the required structure. Utilizing the specificity of a supermolecular interaction, CDs are ideal, connecting moiety for anchoring biomolecules at the PTT’s nanoplatform surface. For example, the heat-inducible promoter-loaded CRISPR/Cas9 plasmid and polyethyleneimine-modified β-CD were conjugated to the NIR-II type’s AuNR surface. Then, guanidyl, capable of promoting deep tissue penetration and Cas9 plasmid delivery, was decorated to AuNR’s surface through an inclusion effect between AD and β-CD. With the help of guanidyl ligands, photothermally-activated immune checkpoint blockade therapy was achieved for reduced tumor metastasis and prolonged immune memory effects [39]. In another report, the surface of a CuS nanocrystal, a kind of metallic compound nanocomposite with photothermal conversion properties at the NIR region, was sequentially modified with a poly(maleic anhydride)-based amphiphilic polymer and β-CD. As β-CD was further conjugated with an AD-modified RGD peptide, the obtained CuS nanocrystal displayed a tumor integrin-targeted PTT under the guidance of photoacoustic imaging [40].

Besides targeting ligands, positively charged biopolymers capable of loading a DNA sequence for combined PTT and gene therapy can also be introduced by CDs’ supermolecular interaction. After loading quantum dot and DOX at its mesoporous silica shell, polycation poly(glycidyl methacrylate)-modified β-CD was coated at the surface of the core-shell AuNR. The β-CD not only acted as a gatekeeper for the triggered DOX release but also introduced polycation for antioncogene p53 loading and AuNR in vivo stabilization. The obtained hetero-AuNR can perform synergistic photothermal-chemo-gene therapy with enhanced specificity under the guidance of tomography, fluorescence, and PA imaging [41]. Zhao et al. prepared a 1-D nanofibril prepared from dextran and Fe_3_O_4_ and decorated poly(glycidyl methacrylate)-β-CD at the nanofibril surface through a β-CD and AD interaction. Compared to its spherical counterpart, the 1-D nanofibril exhibited a significantly accelerated cell uptake rate under the magnetic field treatment, which further led to substantially enhanced gene transfection efficiency. Moreover, the 1-D nanofibril would rotate under an alternating magnetic field, which can cause cancer cell destruction due to the rotary shear force. Therefore, combined gene/photothermal/magnetolytic therapy with synergistic treatment against a 4T1 tumor was observed for this 1-D nanofibril [42].

## 3. CD-Based Nanoplatform for PDT and Its Combined Therapies

PDT relies on the cytotoxicity of ROS generated by photosensitizers (PS). Ideal PS should accumulate in tumor tissues and possess a high photostability. However, most PS display low bioavailability and a hampered ROS-producing efficacy as a result of their hydrophobic nature and potential aggregation in the physiological condition. Furthermore, PDT is limited by hypoxic TME and a short tissue penetration depth of visible light [54,55]. In this section, we first introduce the conjugation of PS with CDs for enhanced bioavailability and photostability. Then, we discuss CDs’ hierarchy self-assembly nanostructure-based PDT nanoplatform for enhanced tumor treatment efficacy (Table 2).

### 3.1. CD and Photosensitizer Conjugation

**Figure 4:**
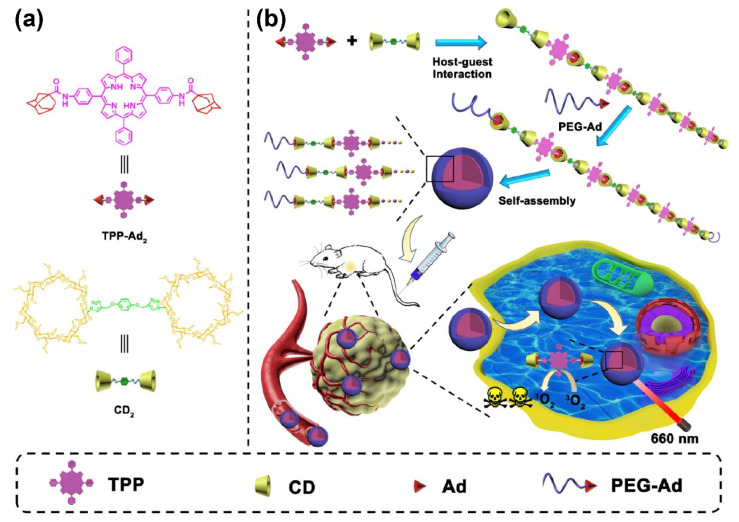
(**a**) The chemical structures of the adamantyl-modified tetraphenyl porphyrin and the cyclodextrin dimer. (**b**) Illustration of the formation of nanoparticles from linear supramolecular polymers with an alternating porphyrin/cyclodextrin structure by the host–guest interaction and self-assembly, and the enhanced PDT process. (Adapted with permission from Ref. [57]. Copyright 2020, Amer. Chemical Soc.).

Porphyrin and its derivatives are the most intensively investigated PS in clinical trials [55]. Due to its cyclic structure formed through the π-π conjugation of four pyrrole rings, porphyrin is quite hydrophobic and displays an aggregation-induced quenching after being intravenously administered. Utilizing the hydrophilic surface of CDs, porphyrin solubility can be promoted without hampering its ROS-generating ability [56,70,71,72,73]. Ikeda et al. reported that porphyrin derivatives and trimethyl-β-CD could readily form a water-soluble supermolecular complex. Importantly, aniline- and phenol- substitutions of β-CD were found to have promoted PDT’s performance against the HeLa cell due to their electrostatically accelerated cell internalization rate [70]. Another group found that the inclusion of a porphyrin derivative, (5,10,15,20-tetrakis(3-hydroxyphenyl) chlorin (mTHPC), within trimethyl-β-CD can strongly restrict the rotation of mTHPC’s phenol rings, which further led to an elevated fluorescence quantum yield. As a result, the fluorescence of the mTHPC and trimethyl-β-CD complex can be used to monitor the in vitro delivery and release of mTHPC [71]. In another work, β-CD modified with the hydrophilic group 2-hydroxypropyl was observed to promote the water solubility and fluorescence quantum yield of chlorophyll a, which is a highly hydrophobic porphyrin derivative extracted from plants. In consequence, the chlorophyll-encapsulated 2-hydroxypropyl-β-CD displayed a higher cellular uptake rate and enhanced PDT’s performance against HT-29 colorectal cancer cells [56]. Besides being directly encapsulated within CDs, PS were also modified with moieties having a strong interaction with the CDs for indirect supermolecular inclusion [57,58,74]. Recently, one group reported that the bis-AD-modified porphyrin and β-CD dimer could be used to prepare an ABA tri-block type amphiphilic supramolecular polymer with an AD- modified PEG at both terminals. The obtained supermolecular polymer can self-assemble in water to form a spherical porphyrin-loaded nanostructure for enhanced porphyrin biocompatibility and solubility (Figure 4). In addition, the alternating structure can reduce the aggregation-induced quenching of porphyrin by the steric hindrance of β-CD, which significantly promotes the in vivo PDT’s performance of porphyrin [57]. 

### 3.2. CD-Based Nanoplatform for Responsive PDT

^1^O_2_ produced by the direct energy transfer from the PS triplet state to oxygen is one of the most active ROS and can oxidate a variety of biomolecules, such as lipids, polysaccharides, and amino acids, to induce cytotoxicity. Therefore, ^1^O_2_ can irreversibly damage cell membranes and subcellular structures, such as the mitochondria and Golgi apparatus and plays a major role in tumor inhibition under PDT treatment [54,55]. However, ^1^O_2_ displays an extremely low maxim active radium (<20 nm), indicating that the accumulation of PS in tumor tissue is a prerequisite for a highly specific and efficient PDT. To increase PS’ delivery specificity, Yoon et al. conjugated γ-CD with PEG and a pH-responsive moiety, 3-(diethylamino)propylamine (DEAP). Due to the hydrophobicity of the DEAP in the neutral solution, the modified γ-cyclodextrin self-assembled in water to form a porous nanoparticle with the ability to encapsulate PS chlorin e6 (Ce6) through supermolecular interactions. After reaching the tumor tissue, the DEAP moiety was dramatically protonated in the acid TME, leading to the disassembly of porous nanostructures and the specific release of Ce6 for an enhanced PDT against the MDA-MB-231 tumor [59]. In another work, AuNP’s surface was coated with a primary amine-modified γ-CD, which was further electrostatically complexed with a 2,3-dimethylmaleic acid-modified γ-CD. In the acid TME, the complex at AuNP’s surface was destabilized due to the decoupling of the 2,3-dimethylmaleic acid. As a result, the cell uptake rate of positively charged AuNP was significantly accelerated for enhanced PTT and highly specific PDT [74]. 

**Figure 5:**
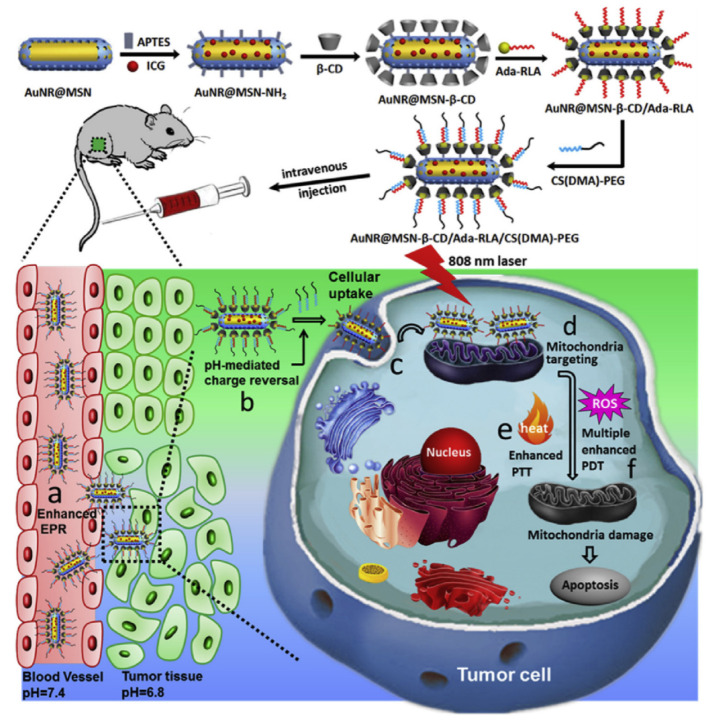
Schematic illustration of the preparation of the AuNR@MSN-based multifunctional nanoplatform and its work process in vivo: (**a**) tumor accumulation of nanosystem via the EPR effect; (**b**) removal of PEG-modified chitosan by slight acidity and exposure of the functional [RLARLAR]_2_ peptide; (**c**) peptide-mediated internalization and (**d**) mitochondrial targeting; (**e**) NIR light-mediated enhanced photothermal and (**f**) photodynamic therapy. (Adapted with permission from Ref. [62]. Copyright 2018, Elsevier).

The host–guest interaction of CDs makes them ideal candidates for developing anti-cancer nanoplatforms with programmed responsiveness [61,62,75]. In a recent report, the pH-responsive molecule benzimidazole, capable of supermolecularly conjugating with β-CD, was introduced to the side chain of PEG-b-polylysine. Then, an obtained polypeptide was sequentially mixed with the (2-hydroxypropyl)-β-CD and Ce6-modified α-CD to obtain polyrotaxane as the hydrophobic segment and (2-hydroxypropyl)-β-CD-capped polylysine as the hydrophilic segment for forming negatively charged supermolecular nano-assemblies in water. The negative surface charge of the obtained nanoassembly can prevent the undesired retention of normal tissues in the circulation system, while benzimidazole and β-CD detached from the polylysine backbone to convert the nanoassembly surface to a positive charge and promote its cell internalization rate [61]. As Ce6 was linked to α-CD through disulfide bonds, the loaded Ce6 could be released for an enhanced PDT after being internalized by cancer cells. Indocyanine green, as an FDA-approved PS for tumor PDT therapy, was loaded to the porous silica layer of AuNR@MSN (Figure 5). The pores of MSN were covalently capped by the CDs as a gatekeeper to prevent the leaky release of indocyanine green, which was further conjugated with an AD-modified peptide possessing membrane penetration and mitochondrion targeting abilities [62]. Then, the charge-reversible polymer 2,3-dimethylmaleic anhydride and PEG-modified chitosan were introduced to AuNR@MSN’s surface through its electronic interaction with the peptide. Once reaching the tumor tissue, the acid TME could dissociate the modified chitosan from the nanoparticle surface to re-expose the targeting peptide, which could enhance the binding of AuNR@MSN to mitochondrion for a highly efficient PDT treatment.

Due to the high reactivity of ROS, there are a variety of moieties that are sensitive to ROS and can undergo bond cleavage or hydrophilicity transfer under ROS treatment. Nanostructures with ROS-responsive moieties as crosslinkers or hydrophobic blocks display photodynamic-triggered cargo release profiles, which were heavily applied to develop nanoplatforms with a combined PDT and chemotherapy. Jia et al. covalently decorated β-CD with PEG and a 4-(hydroxymethyl) phenylboronic acid pinacol ester, which was used to form DOX and a purpurin 18-loaded nanoassembly. Under 660 nm light irradiation, ROS, produced by purpurin 18, not only caused tumor tissue damage but also induced the hydrophobic to the hydrophilic transfer of phenylboronic acid to trigger DOX’s release. This dual drug-loaded micelle exhibited good permeability within solid tumor tissue under confocal microscopy analysis and displayed a synergistic PDT and chemotherapy against the 4T1 tumor on a nude mouse under 660 nm light irradiation for 5 min [63]. Tian et al. mixed a thioketal-linked AD dimer, triphenylphosphine-modified AD, PEG-modified AD, and tetra β-CD-modified porphyrin in water to form a DOX-loaded self-assemble nanostructure with mitochondria targeting ability. Due to the cleavage of thioketal under the ROS treatment, the in vivo distribution of DOX and porphyrin can be readily adjusted by 660 nm light irradiation to achieve highly specific combinational PDT and chemotherapy [64].

**Figure 6:**
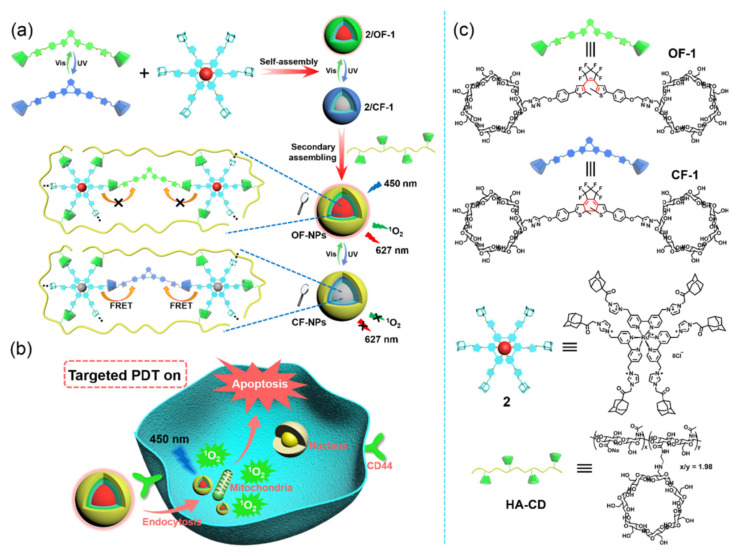
(**a**) Schematic diagram of the formation of CD secondary assemblies with the controllable ^1^O_2_ generation ability and (**b**) their application for targeted PDT; (**c**) the chemical structures of diarylethene-bridged CD in its ring-opened form OF-1 and ring-closed form CF-1; AD polypyridyl ruthenium photosensitizer (2) and β-CD-grafted hyaluronic acid. (Adapted with permission from Ref. [65]. Copyright 2020, Amer. Chemical Soc.).

Another research direction for enhancing PDT specificity has been focused on developing PS with stimuli activable ROS and/or ^1^O_2_ generating ability. Dai et al. synthesized diarylethene-bridged β-CD dimer and AD-modified PS polypyridyl ruthenium, which could form a supermolecular nanoassembly in water with β-CD grafted to HA. Under 245 nm light irradiation, the diarylethene moiety was transformed to a closed ring structure, which triggered the energy transfer from polypyridyl ruthenium to diarylethene and quenched its ^1^O_2_ generating ability. The closed ring structure can be reopened under a long wavelength (>490 nm) light irradiation to activate ^1^O_2_ generation for the tumor PDT treatment (Figure 6). This reversible PDT nanoplatform, in its activated state, displayed an A549 cancer cell inhibition rate 4.4 times higher than its quenched state and is suitable for minimizing inadvertent PS activation during PDT treatment [65].

### 3.3. CD-Based Nanoplatform for Multifunctional PDT

**Figure 7:**
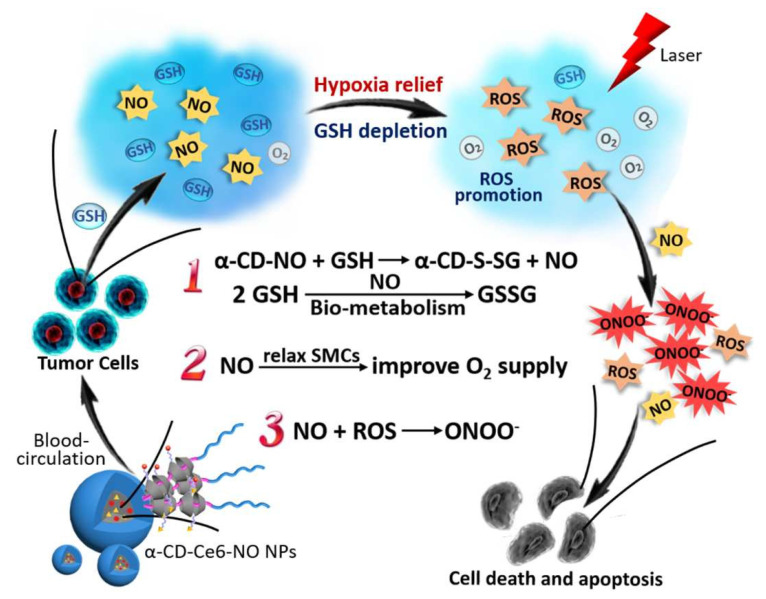
Multiple synergistic effects between NO and PDT generated from the supramolecular nanoparticles α-CD-Ce6-NO nanoparticles to improve therapeutic efficacy. (Adapted with permission from Ref. [68]. Copyright 2018, Elsevier).

In this section, we introduce the CD-based PDT multifunctional nanoplatform dedicated to removing PDT obstacles, such as tumor hypoxia and insufficient light penetration depth. As PDT consumes O_2_ to produce ROS during treatment, the hypoxia TME dramatically restricted the application of PDT tumor therapy. To alleviate tumor hypoxia during PDT, PEG-modified Cu_2−x_Se nanoparticles were decorated at the Ce6-loaded β-CD surface. Utilizing the Fenton reaction between Cu^+^ and H_2_O_2_ to produce O_2_, the abundant H_2_O_2_ in TME acted as an O_2_ reservoir to promote the ROS’ generation during PDT treatment. Furthermore, the Fenton reaction can shift the sensitive wavelength of Ce6 to the NIR region and generate a large quantity of ROS with the help of Ce6 for potential deep tumor treatment. An in vivo experiment revealed that those Cu^+^- and Ce6-generated ROS not only prohibited the primary tumor growth but also suppressed tumor metastasis due to the ROS-induced immunogenic cell death [66]. Ferrocence is another frequently used Fenton reaction agent and can be encapsulated within the β-CD cavity through a host–guest interaction. Qin et al. prepared ferrocence and a PEG-modified FFVLG_3_C peptide, which further formed linear supermolecular nanostructures with Ce6-modified β-CD. The amphiphilic linear supermolecular structure was self-assembled to a spherical micelle with enhanced permeability and retention effect-induced passive tumor targeting. Under 650 nm light irradiation, ferrocence can be oxidized to ferrocence^+^ by Ce6-generated ROS and dissociate from the β-CD cavity. Therefore, the micelle would reshape to the ferrocence-loaded peptide nanofibril with enhanced tumor retention and a Ce6-loaded smaller micelle with deeper tissue penetration, respectively. A further in vivo experiment showed that the ferrocence accumulated in tumor tissues could efficiently relieve tumor hypoxia to allow PDT with a positive feedback loop [67]. As the cancer cells overexpressed glutathione, the ROS’ consumption by intracellular glutathione seriously hampered PDT’s therapeutic efficacy. NO is a bio-signal molecule involving a variety of physiological and pathological processes. Like ROS, NO, as a kind of oxidant, can damage mitochondria and the DNA structure and can react with glutathione to promote PDT’s efficacy. More importantly, NO can cause tumor vasculature relaxation, leading to an elevated blood flow rate and O_2_ supply. Inspired by this, Deng et al. conjugated α-CD with an NO generator s-nitrosothiol and Ce6, respectively (Figure 7). The obtained α-CD-Ce6 and α-CD-NO can form a supermolecular micelle with PEG-b-poly(2-methacryloyloxyethyl phosphorylcholine). After being internalized by cancer cells, the s-nitrosothiol moiety was activated by intracellular glutathione to generate a large quantity of NO, which in turn depleted the glutathione and explosively amplified PDT’s efficacy [68]. 

Despite a great effort in optimizing its performance in PDT, most PS under clinical trials are only sensitive to visible light. Due to the scattering and absorption of visible light by the human body, visible light-sensitive PS are not suitable for deeper tumor treatments. To overcome this, upconverting a nanoparticle capable of converting NIR light to visible light was co-loaded with PS. Yao et al. synthesized an upconverting nanoparticle with a layer of mesoporous silica at its surface. After loading camptothecin within the mesoporous silica layer, PS 1,8-dihydroxy-3-methylanthraquinone were loaded at the silica layer’s surface through a UV-activable bond. Then, lactobionic acid and PEG-modified β-CD were conjugated with 1,8-dihydroxy-3-methylanthraquinone through a supermolecular interaction for asialoglycoprotein receptor targeting. Under NIR light irradiation, the dual emissive upconverting nanoparticle can convert 980 nm of light to 360 nm of light, activating camptothecin’s release, and 480 nm of light can trigger ROS’ generation, which could significantly enhance tumor treatment efficacy [69].

## 4. Conclusions and Future Perspectives

CDs can be readily used to promote the photoeffect of tumor phototherapy by constructing an ideal nanoplatform for PTT with enhanced PCE or PDT with an elevated PS bioavailability and quantum yield. In addition, various bioactive cargoes can be loaded within the cavity of CDs with a photothermally- or photodynamically-triggered release profile. As a result, CDs’ hybridization showed great promise in developing tumor phototherapy nanoplatforms in combination with other tumor therapies in a synergistic manner. Particularly, CDs can be used to prepare a tumor phototherapy nanoplatform with responsive morphological change, which can cause the physical disruption of subcellular structures. Furthermore, this type of responsive nanoplatform can be used to achieve a cell membrane and organelle dual-targeting delivery in a sequential manner. 

However, most combinational therapies in this area focus on the combination of chemotherapy with PTT or PDT, and more effort is deserved to investigate CD-based tumor phototherapy nanoplatforms in combination with immunotherapy, as immunotherapy is considered the most promising tumor treatment modality. The long-term toxicity of inorganic PTAs is an unpredictable hurdle for the clinical translation of PTT. Therefore, using CD-based nanoassembly as a nanotemplate would be a promising route to develop a biocompatible PTT nanoplatform. For PDT, porphyrin and its derivatives were the most investigated PS with their excitation wavelength in the visible light region. Thus, there is a great need to developing an NIR type PS and encapsulating the NIR type PS with CDs to enhance their bioavailability would be a hot topic in the coming future. Overall, CDs’ hybridization can dramatically promote the anti-tumor efficacy of tumor phototherapy and is especially valuable in developing a multifunctional phototherapy nanoplatform to conquer current obstacles in tumor treatment. 

## Figures and Tables

**Table 1:** A summary of the CD-based PTT nanoplatform.

Guest Molecule	PTA Type	Combined Therapy	Cell or Tumor Type	References
PEG	Gold nanoshells	Chemotherapy	HeLa cell	[25]
None	Gold nanotriangles, gold nanoflowers	None	A673 cell	[26]
Pyridine-2-imine	AuNP aggregation	None	MCF-7 tumor.	[27]
Azobenzene	GO	Mitochondrial physical disruption	S180 tumor	[28]
Cyclohexylalanine	AuNR	Mitochondrial physical disruption	A549 tumor	[29]
Camptothecin	GO	Chemotherapy	BEL-7402 tumor	[30]
Paclitaxel	Polydopamine	Chemotherapy	MDA-MB-231 cell	[31]
10-hydroxy camptothecin	Palladium nanosheet	Chemotherapy	H-22 tumor	[32]
AD	Mesoporous platinum nanoparticle	Chemotherapy	MCF-7 tumor	[33]
None	Quaterrylene bisimide derivative	None	HepG2 tumor	[34]
PEG	Perylene diimide	Chemotherapy	HeLa tumor, A549 tumor	[35]
AD	AuNR	Chemotherapy	S180 tumor	[36]
PEG	AuNR	Chemotherapy	4T1 tumor	[37]
PEG	AuNR	Chemotherapy	MDA-MB-231 tumor	[38]
AD	AuNR	Immunotherapy, genetherapy	B16-F10 tumor	[39]
AD	Copper sulfide nanoparticles	Chemotherapy	HeLa cell	[40]
AD	AuNR	Chemotherapy, genetherapy	C-6 tumor	[41]
AD	Fe_3_O_4_ nanoparticle	Genetherapy, magnetolytic therapy	4T1 tumor	[42]

**Table 2:** A summary of the CD-based PDT nanoplatform.

Guest Molecule	PS Type	Combined Therapy	Cell or Tumor Type	References
Chlorophyll a	Chlorophyll a	None	HT-29 cell	[56]
AD	Porphyrin	None	4T1 tumor	[57]
AD	Porphyrin	None	4T1 tumor	[58]
Ce6	Ce6	None	MDA-MB-231 cell	[59]
Ce6	Ce6	PTT	MDA-MB-231 tumor	[60]
PEG	Ce6	None	LM3 tumor	[61]
AD	Indocyanine green	PTT	MCF-7 tumor	[62]
None	Purpurin 18	Chemotherapy	4T1 tumor	[63]
AD	Porphyrin	Chemotherapy	4T1 tumor	[64]
AD	Polypyridyl ruthenium	None	A549 cell	[65]
Ce6	Ce6	Immunotherapy	4T1 tumor	[66]
ferrocence	Ce6	None	4T1 tumor	[67]
PEG	Ce6	None	MCF-7 tumor	[68]
Camptothecin	Ce6	Chemotherapy	HepG2 tumor	[69]

## Data Availability

Not Applicable.

## Abbreviations

AD: adamantane; AuNP: gold nanoparticles; AuNR: gold nanorods; Ce6: chlorin e6; CD: cyclodextrins; DEAP: 3-(diethylamino)propylamine; DOX: doxorubicin; GO: graphene oxide; LSPR: localized surface plasmon resonance; NIR: near infra-red; MSN: mesoporous silica nanoparticle; mTHPC: (5,10,15,20-tetrakis(3-hydroxyphenyl) chlorin; PCE: photothermal conversion efficiency; PEG-b-PC: poly(ethylene glycol)-b-polycysteine; PDT: photodynamic therapy; PTA: photothermal agents; PTT: photothermal therapy; PS: photosensitizers; ROS: reactive oxygen species; TME: tumor microenvironment.
